# Letter regarding “Developing a predictive model for spinal shock in dogs with spinal cord injury”

**DOI:** 10.1111/jvim.16631

**Published:** 2023-01-23

**Authors:** Charles O. Cummings

**Affiliations:** ^1^ Tufts Clinical and Translational Science Institute, Tufts Medical Center Boston Massachusetts USA

Dear Editor,

In a recent Journal of Veterinary Internal Medicine article titled “Developing a predictive model for spinal shock in dogs with spinal cord injury,” the authors create a clinical predictive model (CPM) that estimates the probability that dogs with reduced pelvic limb reflexes have a T3‐L3 myelopathy with concurrent spinal shock vs a T4‐S3 myelopathy.^1^ The model is elegant in that it uses 4 readily assessed parameters: the natural logarithm of weight in kilograms, the natural logarithm of the duration of clinical signs in hours, the presence or absence of paraplegia, and the presence or absence of decreased pelvic limb tone. The model has the potential to be useful to clinicians because it could help them to ensure that they include the affected area (ie, T3‐L3) in diagnostic imaging.^1^ While this is a promising CPM seeking to predict an interesting and important outcome, there are several considerations to be made before this or similar CPMs should see widespread clinical use.

One important criterion to consider when appraising a CPM is whether or not the model has been validated. Model validation aims to assess how well a CPM performs using data that were not used to develop the model. Internal validation uses the same cohort of data on which the model was developed; this can be done by either splitting the dataset into training vs testing sets, or, preferably, by performing k‐fold cross‐validations or bootstrap validations. External validation evaluates how the model performs with data from a different time period, geographic location, or care setting from the model development cohort. External validation is generally preferable to internal validation because models that perform well in external validation datasets are thought to be more generalizable. In the case of this canine spinal shock model, the authors appear to perform a split dataset internal validation with training set of 64 dogs and a test set of 8 dogs.

Possibly the biggest consideration, however, is the risk of bias (ROB) in a model. Bias is quite common in CPMs and occurs when predicted results systematically deviate from the truth.^2^ Risk of bias assessments are increasingly performed for appraisal of CPMs in human medicine using the Prediction model Risk Of Bias ASsessment Tool (PROBAST) in its long or short form.^3^, ^4^ The population on which the model is developed, the predictors used, and the outcomes assessed can all result in a model being at high ROB. Most often, however, it is analytical choices that result in a model being at high ROB.^4^ Importantly, just because a model is at high ROB does not mean that it has no clinical utility.^2^ In a recent systematic review of CPMs in equine medicine, the authors found 90/90 models to be at high ROB, which is consistent with similarly high rates found in systematic reviews of CPMs in the human medical literature.^2^, ^5^ Clearly, there is an extremely high bar to clear for a CPM to be considered at low risk of bias, and some causes of a high ROB, such as low patient numbers compared to candidate predictor variables, will always be challenging to avoid. Nevertheless, it is important to be open about potential risks of bias when writing about a new CPM so that clinicians are better suited to appraise the model and how much they believe in its predictions. In this CPM of spinal shock in dogs, there are several analytical choices that place the model at high ROB. These include exclusion of dogs with missing data (as opposed to multiple imputation of missing data), univariate screening of predictor variables (can fail to include variables that are associated with the outcome in the context of other variables), insufficient number of events per candidate variable (~1.1 events/per candidate predictor, as opposed to 10 events/candidate predictor, an imperfect but commonly used heuristic), and a lack of correction for model overfitting/optimism. While none of these issues is necessarily a fatal flaw for the model, the presence of any particular one (or another PROBAST concern) indicates that a model is at high risk of bias.^3^, ^4^

When CPMs are not validated, are validated using small internal validations (as seen here), or are at high ROB, model performance metrics are typically overoptimistic, meaning that the model will likely perform worse in new populations. In the case of this model of spinal shock in dogs, the authors report an exceptional area under the receiver operating characteristic curve (AUC) of 0.91. This means that for any random pair of animals, one with a T3‐L3 myelopathy and spinal shock and one with an L4‐S3 myelopathy, there is a 91% chance that the dog with the T3‐L3 myelopathy has a higher predicted probability of spinal shock. To put this into context, an AUC of 0.5 indicates that the model discriminates no better than a coin flip, while an AUC of 0.7‐0.8 is considered to be acceptable, 0.8‐0.9 is considered to be excellent, and greater than 0.9 is outstanding.^2^ While we do not yet know how this spinal shock model will perform in an external validation, it is reasonable to assume performance will drop off to some degree.

Lastly, some clinicians carefully consider all the above risk of bias or validation‐related points and decide they would like to try to externally validate a CPM in their cohort of affected dogs to see if it could be useful to them. Yet, many predictive models fail to see use because there is no simple tool with which to apply the model.^2^, ^6^, ^7^ For the current model, the authors do well to provide the full model equation and even demonstrate the equation's utility with a couple clinical vignettes.^1^ Unfortunately, however, they do not provide a simple tool like a sum score or spreadsheet that would allow others to quickly perform model calculations for dogs they are managing.^7^ This oversight could prevent other investigators and clinicians from using the model.

Transparency in reporting veterinary CPMs and their potential limitations, including high ROB and overoptimism of performance estimates, as well as provision of tools to simplify external validation (and eventually simplify clinical use) are vitally important for CPMs to move from the page to the clinic.^3^, ^6^, ^7^, ^8^ I believe that the predictive model developed by McBride et al has the potential to be a useful clinical tool. This letter can help clinicians critically appraise this and other predictive models and potentially validate them in their own patient populations.

## References

[1] McBride R , Parker E , Garabed RB , et al. Developing a predictive model for spinal shock in dogs with spinal cord injury. J Vet Int Med. 2022;36:663‐671. 10.1111/jvim.16352 PMC8965241 35001437

[2] Cummings CO , Krucik DDR , Price E . Clinical predictive models in equine medicine: A systematic review. Equine Vet J. 2022. 10.1111/evj.13880 PMC10073351 36199162

[3] Moons KGM , Wolff RF , Riley RD , et al. PROBAST: a tool to assess risk of bias and applicability of prediction model studies: explanation and elaboration. Ann Intern Med. 2019;170:W1. 30596876 10.7326/M18-1377

[4] Venema E , Wessler BS , Paulus JK , et al. Large‐scale validation of the prediction model risk of bias assessment tool (PROBAST) using a short form: high risk of bias models show poorer discrimination. J Clin Epidemiol. 2021;138:32‐39. 34175377 10.1016/j.jclinepi.2021.06.017

[5] Wynants L , Calster BV , Collins GS , et al. Prediction models for diagnosis and prognosis of COVID‐19: systematic review and critical appraisal. BMJ. 2020;369:m1328. 32265220 10.1136/bmj.m1328 PMC7222643

[6] Wyatt JC , Altman DG . Commentary: prognostic models: clinically useful or quickly forgotten? BMJ. 1995;311:1539‐1541.

[7] Cummings CO . Letter to the editor: a tool for calculating VetCOT score. J Vet Emerg Crit Care. 2022;32:426. 10.1111/vec.13194 35363939

[8] Moons KGM , Altman DG , Reitsma JB , et al. Transparent reporting of a multivariable prediction model for individual prognosis or diagnosis (TRIPOD): explanation and elaboration. Ann Intern Med. 2015;162:W1‐W73. 25560730 10.7326/M14-0698

